# Rwandan stakeholder perspectives of integrated family planning and HIV services

**DOI:** 10.1002/hpm.2586

**Published:** 2018-07-26

**Authors:** Kristin M. Wall, Roger Bayingana, Rosine Ingabire, Lauren Ahlschlager, Amanda Tichacek, Susan Allen, Etienne Karita

**Affiliations:** ^1^ Rwanda Zambia HIV Research Group, Department of Pathology and Laboratory Medicine, School of Medicine and Hubert Department of Global Health, Rollins School of Public Health Emory University Atlanta GA USA; ^2^ Department of Epidemiology, Rollins School of Public Health, Laney Graduate School Emory University Atlanta GA USA; ^3^ Rwanda Zambia HIV Research Group, Department of Pathology and Laboratory Medicine, School of Medicine and Hubert Department of Global Health, Rollins School of Public Health Emory University Kigali Rwanda

**Keywords:** family planning, HIV, policy change, Rwanda, service integration

## Abstract

The purpose of this qualitative study was to understand the knowledge, attitudes, and practices among key Rwandan policymakers and stakeholders related to family planning (FP) and integrated HIV/FP services. Motivational in‐depth interview format and content was developed after an extensive policy review. A convenience sample of 10 high‐level HIV and FP Rwandan policymakers and stakeholders completed the interview. Stakeholders demonstrated strong foundational knowledge of HIV and FP. Given the choice, stakeholders would allocate more monies to FP and less to HIV than currently distributed. Respondents felt that improved FP method knowledge, especially long‐acting reversible contraception, among clients/couples and providers, was needed to address myths, misconceptions, and biases. The most often cited way to integrate HIV/FP services was development of integrated tools (eg, training materials, data collection tools, and advocacy and policy guidance). We recommend strategies for policy advancement supportive of HIV/FP service integration inclusive of couples and long‐acting reversible contraception methods.

## 1 BACKGROUND

Rwanda is the most densely populated country in Africa, with total fertility rates (TFR) ranging from 3.6 in urban to 4.3 in rural areas.^1^ While the prevalence of modern contraceptive use in reproductive age women in Rwanda has improved dramatically over recent years, increasing from 4% in 2000 to 48% in 2014 to 2015,^1^, ^2^ less than 5% of reproductive age women are using highly effective and cost‐effective long‐acting reversible contraceptives (LARC)—specifically the implant and intrauterine device (IUD).^3^, ^4^

Integrating family planning (FP) and HIV services has broad support from international stakeholders^5^, ^6^, ^7^ to reduce unmet need for contraception, unintended pregnancy, and as a low‐cost perinatal HIV prevention strategy.^8^ Heterosexual cohabiting couples are the largest risk groups for both unintended pregnancy and HIV, and Rwanda is the only country to implement couples' voluntary counseling and testing (CVCT) programs nationwide^9^ as a high impact HIV prevention strategy.^10^

Family planning is among Rwanda's top health policy priorities,^11^ and there is strong political support for HIV and FP service integration, including CVCT and LARC, as demonstrated in the National Guidelines for Comprehensive Care of People Living with HIV in Rwanda^12^ and the National Family Planning Policy which detail integration of FP with HIV services.^13^ However, the government acknowledges that current policies have neither resulted in implementation of HIV/FP service models nationwide nor promotion of LARC methods.^11^, ^12^, ^13^

The Rwanda Zambia HIV Research Group (RZHRG), established in Kigali, Rwanda, in 1986, successfully supported the government to establish CVCT as a national standard of care in all government antenatal clinics through research, outreach, policy, and advocacy efforts.^9^ Using the same comprehensive approach, RZHRG is now establishing LARC provision as standard of care in FP programs, and integrating FP services with couple‐focused HIV testing programs. To ensure successful integration, efforts must move past the clinic staff and client levels to engage stakeholders at the policymaker and funding agency levels.

The purpose of this qualitative study was to understand the knowledge, attitudes, and practices of key policymakers and stakeholders in Rwanda related to HIV prevention, CVCT, LARC, and integrated HIV/FP services via motivational in‐depth interviews. The ultimate goal of this work is to build consensus and secure commitment for future policies supporting an integrated HIV and FP service model including CVCT and LARCs.

## 2 METHODS

### 2.1 Participants

We recruited senior policymakers and stakeholders to complete in‐depth motivational interviews using structured mixed‐methods (including both open‐ended and close‐ended response) interviewer‐administered questionnaires. Participants included government officials, leaders of international and bilateral funding agencies, and HIV and/or FP implementing partners. Eligible candidates were identified through the Ministry of Health (MoH) Maternal and Child Health Division, the relevant units of the Rwanda Biomedical Center (the MoH entity responsible for HIV and infectious disease activities), and the Rwanda FP Technical Working Group, which is comprised of international and bilateral funding agency representatives as well as key government staff and implementing partners. Invitations to potential participants were delivered and followed up with phone calls to set interview appointments. Interviews were conducted between April 2014 and June 2014 until saturation was achieved around key ideas.^14^ Motivational interviewing (an interviewing style with demonstrated efficacy in numerous randomized trials which focuses on “reflective listening, shared decision‐making, and eliciting change talk”^15^) was used as the framework to solicit informed opinions from the respondents. Identifiers were not linked to responses.

### 2.2 Motivational in‐depth interview methods

Face‐to‐face motivational in‐depth interviews (IDIs) were conducted in English, French, or Kinyarwanda by coauthor and physician RB in Kigali. The interviewer received training in motivational IDI techniques, including avoiding leading questions and using impartial language and body language.^16^ The interviews were structured as mixed‐methods questionnaires administered using a laptop which included both open‐ended and coded response questions that were recorded by the questionnaire facilitator. The interviews lasted less than 1 hour, and participants were not offered incentives because of their very senior status.

### 2.3 Questionnaire development and content

To develop the questionnaire, coauthor RI reviewed Rwandan HIV and FP policy documents currently in use by the MoH. The documents included the Rwandan FP Strategic Plan 2012 to 2016,^11^ the 2012 National FP Policy which details integration of FP with HIV/AIDS services,^13^ and 2011 National Guidelines for Comprehensive Care of People Living with HIV in Rwanda.^12^ This policy review showed that though FP and HIV are top health priorities in Rwanda, the government acknowledges that the current method mix does not address the fertility goals of many women, that use of short‐acting FP methods results in unintended pregnancies, that current promotion strategies do not address low LARC use, that male involvement is needed in FP as well as HIV prevention, and that successful models for FP and HIV service integration are needed. The review highlighted the lack of specific plans (beyond broad endorsement) for integrating HIV and FP efforts, with documents in each domain referring to the other in general terms. The policy review informed the development of the structured questionnaire. Questionnaires were pilot tested by coauthor RI in February to March 2013 with the in‐charge of the prevention of mother‐to‐child transmission department at the Rwanda Biomedical Center and 4 additional senior RZHRG personnel and were revised. To safeguard confidentiality, identifiers were not included on questionnaires.

Stakeholder professional background/organizational involvement was assessed via open‐ended questions about professional background or current professional activities in HIV or FP programs. After respondents were shown a pie chart detailing the US Bilateral Foreign Assistance allocations to HIV and FP in Rwanda, stakeholders were asked what percentage of the budget they would allocate to HIV and FP. Participants also selected the data sources their organizations used to make decisions about HIV program funding allocations.

Knowledge of HIV epidemiology and prevention strategies in Rwanda was assessed. Participants ranked the top 3 sources of new HIV infections in Rwanda, discussed where CVCT could be offered to reach larger audiences, and selected ways in which serodiscordant couples (1 partner HIV+ and the other HIV−) could protect against HIV transmission. A closed‐ended question ascertained how important respondents felt cost considerations should be when deciding which HIV prevention strategies to implement. After respondents were presented with a pie chart detailing the allocation of HIV aid money in 8 PEPFAR priority countries as well as a discussion of the costs of HIV interventions including antiretroviral therapy (ART) and CVCT, they were asked to allocate the HIV budget as if they were the Minister of Health.

Respondents were then asked about their knowledge of demography and FP in Rwanda. After stakeholders discussed data on current TFR and economic poverty trends in Rwanda, the questionnaire asked stakeholders' views on the relationship between TFR and economic development and discussed a reasonable TFR to achieve in the next 5 years in Rwanda. Next, stakeholders selected factors influencing TFR in Rwanda and ranked a list of contraceptive methods in best options for Rwanda. An open‐ended question assessed responders' thoughts on obstacles to increasing LARC uptake. Respondents were also asked to select from a list of options to enhance LARC uptake, including modification to Rwanda's performance‐based financing system which currently reimburses facilities for new contraceptive method users.^17^, ^18^, ^19^

A final set of questions addressed integration of HIV and FP services (Table 4). First, a closed‐ended question assessed if respondents believed that providing LARC to HIV discordant couples may affect condom use. After stakeholders discussed facts about LARC and condom use, a series of open‐ended questions were asked: How can we ensure sustainable integration of HIV and FP services in the health care system? What are your opinions on offering CVCT/VCT services to people who are coming for FP as a strategy for the integration of HIV/FP? What are your opinions on offering a full range of FP options to people who are coming for HIV services as an integration strategy? What are your opinions on discussing issues related to child spacing and FP options in antenatal clinics when both women and men come together to get tested for HIV? What are your opinions on promoting and offering CVCT/FP services in infant vaccination programs?

### 2.4 Data analysis

Deidentified questionnaire data were entered on‐site in Kigali into a Microsoft Access database for quality control, cleaning, and analysis. Responses from closed‐ended questions were described using counts and percentages. For open‐ended questions, thematic qualitative data analysis methods were applied, and a quantitative summary of all participant responses is presented because of the small sample size.

### 2.5 Ethical approval

These interviews were conducted with senior members of government, donor, and implementing agencies who consented verbally to participate. In this context, participant reimbursement would not be appropriate. No identifiers were recorded.

## 3 RESULTS

### 3.1 Stakeholder organizational characteristics, sources of information used, and preliminary budget allocations for HIV and FP (Table 1)

**Table 1:** Stakeholder organizational characteristics, sources of information used, and preliminary budget allocations for HIV and family planning

Which HIV Activities, If Any, Is Your Organization Involved In?	N	%
Prevention	9	90%
Training	8	80%
PMTCT	7	70%
Technical assistance	7	70%
VCT	6	60%
Policy	6	60%
Funding	5	50%
Treatment	5	50%
Blood safety	1	10%
Laboratory services	0	0%
Which family planning activities, if any, is your organization currently involved in?^a^	N	%
Training	9	100%
Policy	7	78%
Funding	6	67%
Technical assistance	6	67%
Service delivery	4	44%
Promotion, social	4	44%
Commodities	3	33%
Marketing	0	0%
If you were the minister of health of Rwanda, taking all funding sources together, what percentage would you allocate to? (Open‐ended)^b^	Average	Range
Percentage allocated to HIV (0%‐100%)	37.5%	30–60%
Percentage allocated to family planning (0%‐100%)	22.5%	10–40%
Which data sources does your organization use when making decisions about HIV program funding allocation?	N	%
Rwanda demographics health and survey	8	80%
Health management information system	8	80%
Tracnet data	4	44%
Behavior surveillance survey	4	44%

Percentages calculated accounting for nonresponse in denominators.

Abbreviations: VCT, voluntary HIV counseling and testing; PMTCT, prevention of mother‐to‐child transmission.

a n = 9 respondents.

b n = 6 respondents (4 stated that they would need more information to answer the question).

All 10 stakeholders interviewed reported that their organization had actively participated in the formulation of the HIV/AIDS National Strategic Plan including strategy formation, development, costing, implementation, and review. All 10 worked on HIV, specifically in areas of prevention (9), training (8), prevention of mother‐to‐child transmission (7), and technical assistance (7). Other common areas of experience included VCT, policy, funding, and treatment.

Nine respondents' organizations had been involved in the formulation of Rwanda's Family Planning Policy and its 5‐year National Strategic Plan. Their involvement in these policy documents included inception, formulation, strategy development, writing, and review. Most reported their organizations' FP efforts as training (9), policy (7), funding (6), and technical assistance (6).

After stakeholders were presented with the current US Bilateral Foreign Assistance budget allocations (within which HIV received 64% of resources and FP 7%), respondents stated they would allocate on average 37.5% (range 30%‐60%) to HIV and 22.5% (range 10%‐40%) to FP. Most stakeholders reported utilizing data from the Rwanda DHS (8) and HMIS (8) when making funding allocation decisions for HIV programs. Stakeholders also confirmed using Tracnet and behavioral surveillance survey data (4 each) as well as other surveys.

### 3.2 Stakeholder knowledge of HIV epidemiology and prevention strategies in Rwanda (Table 2)

**Table 2:** Stakeholder knowledge of HIV epidemiology and prevention strategies in Rwanda

Please Rank the Top 3 Main Sources of New HIV Infections in Rwanda?^a^	N	%
Sex workers	9	90%
Married adults	6	60%
Men who have sex with men	5	50%
Truck drivers	5	50%
Single adults	2	20%
Youth	2	20%
Men in uniform	1	10%
Prisoners	0	0%
Where else do you think CVCT could be offered to reach a larger audience?		
Community‐based/mobile	10	100%
Premarital VCT	8	80%
Family planning	6	60%
In patient	4	40%
Infant vaccination clinics	3	30%
Out patient	2	20%
None of the above	0	0%
How can a couple with 1 HIV+ and 1 HIV− partner (“discordant couple”) prevent transmission within their marriage?		
Male condom	10	100%
ART for HIV+ partners	8	80%
Female condoms	3	30%
PreP for HIV− partners	1	10%
Vaginal product containing ART	1	10%
Abstinence	0	0%
PEP for HIV− partners	0	0%
How important should cost considerations be when deciding what HIV prevention strategies to implement?		
Critically or very important	8	80%
Somewhat important	1	10%
Minimal	0	0%
Irrelevant	1	10%

Abbreviations: CVCT, couples' voluntary HIV counseling and testing; WHO, World Health Organization; ART, antiretroviral treatment; PreP, pre‐exposure prophylaxis; PEP, post‐exposure prophylaxis.

a N reported are categories selected overall (no ranking).

Eight stakeholders ranked the top 3 sources of new HIV infections in Rwanda while 2 selected 3 categories without ranking them. Irrespective of ranking, 9 respondents selected female sex workers, 6 selected married adults, 5 selected MSM, and 5 selected truck drivers.

All 10 suggested that CVCT could be offered during community‐based/mobile testing, 8 mentioned premarital testing, and respondents also discussed clinic‐based services including FP, inpatient wards, outpatient clinics, and infant vaccination.

When asked how a HIV discordant couple could prevent transmission within their partnership, all 10 cited male condoms and 8 cited ART for the HIV+ partner. One each cited pre‐exposure prophylaxis and vaginal products containing ART. None cited abstinence or post‐exposure prophylaxis.

Eight participants said that cost considerations were critical when deciding which HIV prevention strategies to implement. One respondent thought that cost was somewhat important, and one thought that it was irrelevant.

### 3.3 Stakeholder opinions on HIV resource allocation (Figure 1)

**Figure 1:**
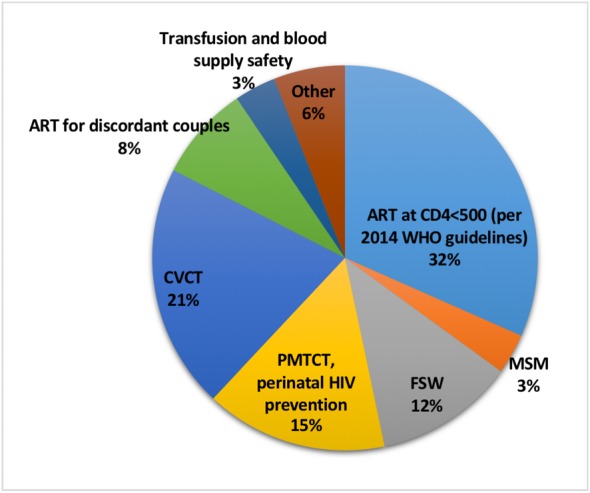
Stakeholder opinions on HIV resource allocation. ART: antiretroviral treatment; CVCT: couples' voluntary HIV counseling and testing; PMTCT: prevention of mother‐to‐child transmission; FSW: female sex worker; MSM: men who have sex with men

Participants would allocate on average 63% of the total budget to preventative services (21% prevention via CVCT, 15% prevention of perinatal HIV, 12% prevention with female sex workers, 8% prevention via ART for discordant couples, 4% prevention via transfusion and blood supply safety, and 3% prevention with MSM) and 32% for ART.

### 3.4 Stakeholder knowledge about demography and FP in Rwanda (Table 3)

**Table 3:** Stakeholder knowledge about demography and family planning in Rwanda

What Relationship Does High Total Fertility Rate (TFR) Have to Economic Development?	N	%
None	0	0%
Slows economic development	9	90%
Improves economic development	1	10%
	Median	Range
What do you think is a reasonable TFR target for Rwanda in the next 5 years? (Open‐ended)	3.0	2.2–2.5
Which of the reasons below do you think affect the TFR in Rwanda? Select all that apply:	N	%
Women and men lack knowledge about contraceptives	8	80%
Health care staff are not adequately trained in family planning	8	80%
Religious beliefs prevent use of contraception	5	50%
Contraceptives are not widely available	3	30%
Couples want large families	2	20%
The MOH has not prioritized family planning	2	20%
Donor governments have not prioritized family planning	2	20%
Which contraceptive methods do you think are best for Rwanda? Please rank them in order of importance (with 1 being the most important):	Average importance	SD
IUD (12 years, reversible)	1.3	0.5
Jadelle (5 years) or implant (3 years) implant (reversible)	1.5	0.5
Injectable contraceptives (every 3 months)	2.9	1.0
Tubal ligation (for the women, permanent)	3.4	1.5
Vasectomy (for men, permanent)	3.4	1.5
Oral contraceptives (“the pill,” 1 per day)	3.5	1.9
What do you think the obstacles are to increasing uptake of IUD and implant methods? (Open‐ended)^a^	N	%
Lack of trained providers to offer LARC	6	67%
Myths/misconceptions	4	44%
Lack of client awareness of the methods	4	44%
Clients choose not to use because of side effects	1	11%
Which of the following interventions do you think are important strategies to enhance uptake of LARC? Please select all that apply:	N	%
Training nurses to insert LARC	10	100%
Educating both men and women about LARC methods	9	90%
Equipping clinics with exam tables, instruments and autoclaves	6	60%
Social marketing of LARC methods	5	50%
Eliminating client payments for LARC methods	5	50%
Increasing performance‐based pay for LARC methods	4	40%
Including men in family planning counseling	1	10%

Abbreviations: TFR, total fertility rate; LARC, long‐acting reversible contraception; IUD, intrauterine device; MOH, Ministry of Health.

a n = 9 respondents.

Nine respondents agreed that Rwanda's high TFR slows economic development. Most stakeholders felt that a TFR of 3.0 (range 2.2‐3.5) would be achievable in the next 5 years (and in open‐ended comments, 4 respondents mentioned the ideal TFR being closer to 2.0 but likely unachievable). Many discussed reasons to be optimistic about future declines in TFR, including upward trends in FP use, education, and empowerment of women. One participant added “… a lot needs to be done to sensitize the communities as to the advantages they would get [from a lower TFR].”

When asked what factors impact the high TFR in Rwanda, 8 cited a lack of knowledge among men and women about contraceptives, 8 cited lack of adequate training among health care staff, and 5 cited religious beliefs. Most (8) did not cite a lack of prioritization by either the MoH or donor governments. Overall, stakeholders ranked the IUD (first choice) and implant (second choice) as the best methods of contraception for Rwanda, with oral contraceptive pills ranking last.

Three primary barriers to adequate uptake of LARC methods emerged in the interviews. Six participants noted that many providers do not know how to properly administer these methods and may prefer administering “easier methods.” One added that providers, “… do not know how to do it so prefer not to talk about it.” Four stakeholders said that clients lack accurate knowledge about the better methods. Finally, 4 stakeholders noted that myths and misconceptions among both clients and providers are issues (examples described included that the Jadelle implant is sometimes seen as a means of abortion, and some believe that IUDs may not be given to women whose hygiene is poor).

All respondents agreed that training nurses to insert LARC was important to enhance uptake, and 9 agreed that educating both men and women on LARC methods was important. Other ways to enhance LARC uptake included providing more LARC supplies (exam tables, instruments, autoclaves) to the clinics (6 respondents), more social marketing (5 respondents), and eliminating client payments for LARC (5 respondents). While 4 stakeholders agreed with increasing performance‐based financing, the Rwandan system for incentivizing high‐quality care in all departments of government health facilities^17^, ^18^ for LARC methods, one mentioned fear of coercion on the part of providers.

### 3.5 Stakeholder opinion on integration of HIV and FP services (Table 4)

**Table 4:** Stakeholder opinion on integration of HIV and family planning services

Discordant Couples Are Advised to Use the IUD/Implant for Effective Prevention of Pregnancy, and to also Use Condoms to Prevent HIV Transmission. In Your Opinion, Would Providing the IUD or the Implant in HIV Discordant Couples Affect Their Condom Use?	N	%
No, condom use will not change	6	60%
Yes, they will be less likely to use condoms	4	40%
Yes, they will be more likely to use condoms	0	0%
How can we ensure a sustainable integration of HIV and FP services in the health care system? (Open‐ended)		
Integrated tools (training materials, data collection, advocacy, policy guidance)	7	70%
Preservice and in‐service training	5	50%
Integrate FP and HIV more broadly within each facility	5	50%
Train health workers to provide all services	4	40%
Integrated funding	2	20%
Use community health workers to promote both	2	20%
What are your opinions on offering CVCT services/VCT services to people who are coming for FP services as a strategy for the integration of HIV/FP services in the health care system? Good idea:	10	100%
What are your opinions on offering the full range of family planning options to people who are coming for HIV services as a strategy for the integration of HIV/FP services in the health care system? Good idea:	10	100%
What are your opinions on discussing issues related to child spacing and family planning options in antenatal clinics when both women and men come together to get tested for HIV? Good idea:	10	100%
Rwanda is the first country that has established CVCT as a standard practice in antenatal clinics. What are your opinions on promoting and offering couples family planning counseling services in infant vaccination programs? Good idea:	9	90%
How far is Rwanda in the implementation of integrated HIV/FP services?		
HIV testing in family planning clinics?		
Well established	1	13%
In progress	7	88%
No plans	0	0%
Family planning in HIV services?		
Well established	2	22%
In progress	7	78%
No plans	0	0%

Abbreviations: IUD, intrauterine device; ART, antiretroviral treatment; CVCT, couples' voluntary HIV counseling and testing; VCT, voluntary HIV counseling and testing; FP, family planning.

Six respondents believed that condom use would not change in discordant couples who use a LARC method, while 4 believed that discordant couples would be less likely to use condoms if provided with LARC. Seven stakeholders discussed the need for integrated training, data collection, advocacy, and policy guidance tools to sustainably integrate HIV and FP services within the Rwandan health care system. The need for preservice as well as in‐service training for both FP and HIV counseling and testing services was mentioned by 5 respondents. Five respondents also mentioned the importance of integrating FP and HIV services more broadly into other services within each health center. As 1 participant noted, “I would like to see a health center where I can go and get services at one stop …. This reduces stigma because everybody comes to that clinic.” Another added, “Awareness that they can get services in one place can motivate the clients to take up this method.” Four stressed training all providers to be multidisciplinary, and the need for dedicated funding for integrated services was mentioned by 2 respondents. Two respondents also noted the important role of community health workers who are able to reach large audiences to efficiently promote both services.

All 10 stakeholders agreed that offering CVCT/VCT to clients coming into a clinic for FP services is a good strategy for integration. All respondents agreed that offering a full range of FP methods to those who are coming for HIV services was a good idea, and one noted that the IUD should be especially emphasized. There was unanimous support for discussing child spacing and other FP options at antenatal clinics when a couple comes in for HIV testing, and 4 participants referred to this as an optimal time to deliver this information.

Nine participants were in favor of promoting and offering CVCT/FP services in infant vaccination programs, with comments that mothers tend not to miss vaccination appointments and that the messaging would be understood “faster when you link the child to good health, social‐economic and FP.” However, 4 participants expressed concerns—3 commented that couples often do not come to infant vaccination appointments together and it is challenging to involve men because of their availability, and 1 participant expressed challenges regarding message saliency within the context of infant vaccinations noting “I don't think you have a focused audience and the retention of the message may be very low.”

Seven participants agreed that integration of HIV testing in FP clinics or integration of FP in HIV clinics was in progress but not well established in Rwanda. Many were of the opinion that while progress is being made, integration may need more central control to be effective. One participant said, “The integration is not at the same level in all health facilities. In some this is well done and in others the gap is still big. We need the same model and increase training and scale up everywhere.” Another added, “There should be a change at central level especially in FP and MCH (maternal and child health). There is no way we can expect results if at the central level we have people who are not cooperative. (We) need to start outreach program first as it was done for HIV.”

## 4 DISCUSSION

Our findings draw from the experience of senior key policymakers engaged in HIV and FP in Rwanda, a sub‐Saharan African country that has had impressive successes in HIV prevention, with prevalence stable at 3% for over a decade among adults age 15 to 49.^1^ While unmet need for contraception and TFR have decreased,^20^ the method mix remains largely limited to oral and injectable contraceptives. Our findings indicate that policymakers believe reallocation of resources will be required to fill gaps in HIV and unplanned pregnancy prevention, and noted several opportunities to increase LARC uptake. To integrate HIV/FP services, stakeholders noted the important role of community health workers, preservice and in‐service provider training in both FP and HIV service provision, integrated training and advocacy tools, and integrated funding and policy guidelines.

Interestingly, stakeholders would allocate on average only 38% of the US Bilateral Foreign Assistance budget to HIV and 23% to FP (as opposed to the 64% and 7% respectively which was the actual allocation at the time). This is not surprising as Rwanda is the most densely populated country in Africa^1^ and the Rwandan government has cited FP as a top policy priority.^11^, ^13^ Most respondents noted that cost considerations were critically important when deciding which HIV prevention interventions to implement, and participants would allocate 63% of the HIV budget for prevention and 32% for treatment (compared to the actual allocation of 21% for prevention and 53% for treatment). While PEPFAR and Global Fund priorities have focused on treatment,^21^, ^22^, ^23^ the perspectives of front‐line decision‐makers appear to prioritize prevention to pre‐empt costly treatment.

Respondents were aware that Rwanda's high TFR slows economic growth, and indicated that improvements in both men's and women's contraceptive knowledge as well as provider training were needed to address myths, misconceptions, and biases. Most stakeholders felt that a TFR of 3.0 was achievable within 5 years and believed that the IUD and implant were the best FP methods for Rwanda to achieve this goal. Half of respondents cited religious beliefs impacting contraception use. Religious leaders, particularly within the Catholic Church, have been a hurdle to contraceptive method use, and roughly 43% of facilities in Rwanda are religious‐affiliated (18% are Catholic). As a result, the Rwandan MoH installed health posts near religious‐affiliated health centers to provide modern contraceptives.^24^ When asked what factors impact the high TFR in Rwanda, most (8) did not cite a lack of prioritization by donor governments, though this conflicted with the substantial difference between how the USG allocated FP funding and how the respondents would allocate that same funding.

Several respondents believed that increasing performance‐based pay specifically for LARC methods to increase uptake was acceptable. The Rwandan performance‐based financing system was introduced in 2002 by international nongovernmental organizations through pilot projects^17^, ^18^ to provide incentives for medical staff to deliver high‐quality services. The system is now managed by the MoH^25^ and is associated with an improvement in nationwide health service uptake.^17^, ^19^ One stakeholder noted concern about possible coercion if LARC methods were reimbursed differently, a concern also raised by USAID.^26^ However, differentiated reimbursement schemes, proportional to the time and costs that clinics incur to provide LARC methods, have been considered and implemented in other locales, for example in Burundi where the payment for new oral and injectable FP users and all return clients was $2.00USD, while for implants/IUDs it was $5.00USD.^27^, ^28^ The importance of male involvement, already a nationwide norm with joint HIV testing of >80% of pregnant women and their partners,^9^ was also mentioned as an important means of increasing effective LARC use.

Integration of HIV and FP services was broadly supported by stakeholders and policymakers. Interestingly, 4 respondents were initially concerned that LARC may decrease condom use in HIV discordant couples, though in fact condom use is more consistent among LARC‐using discordant couples in Rwanda compared with non‐LARC using discordant couples.^29^ Several participants cited antenatal clinics as an optimal venue to discuss HIV testing and FP because husband and wife attend together for CVCT. Stakeholders gave insights around the need for preservice as well as in‐service training for both FP and HIV counseling and testing services. Indeed, the government of Rwanda has prioritized an integration strategy wherein providers are cross‐trained^11^, ^12^, ^13^; however, given the time and training limitations often faced by providers,^30^ referral system strengthening is a potentially less expensive integration method shown to increase linkages between HIV and FP services and decrease unmet need for those services.^31^, ^32^ Strengthening referrals may also allay concerns about message dilution if HIV and FP services are integrated in certain settings, as expressed by 1 stakeholder. Interestingly however, none of our stakeholders raised the idea of referral system strengthening, perhaps reflecting the current government stance on physical integration.^11^, ^12^, ^13^ A more nuanced approach wherein comprehensive HIV and FP counseling is included prior to referral from one service to another might be feasible.

Our respondents noted the role of community health workers in both promotions of integrated HIV/FP services focusing on LARC promotion. In key respondent interviews previously conducted by USAID in Rwanda, strategies for scale‐up of FP programs included training community health workers to provide information on all contraceptive methods and promotion/provision of LARC at health centers and secondary posts, including immediate post‐partum IUDs.^24^ As in other countries, community health workers in Rwanda are now receiving training to provide FP including oral and injectable hormonal contraception within communities.^33^ In Uganda, Village Health Teams integrated HIV counseling and testing into pre‐existing FP services provided by community health workers—they demonstrated that training community health workers who already provide oral and injectable contraceptives to counsel and test for HIV was feasible, acceptable, and could be accomplished without sacrificing to HIV testing quality.^34^ Importantly, the study in Uganda highlighted the need for this model to better incorporate men and couples' counseling.^34^

Stakeholders noted the need for funding and policy guidelines dedicated to integrated services, integrated advocacy messages, and eventual integration into a broader range of services. Such calls to action were made over a decade ago^35^ to coordinate separate sources of funding for National AIDS Control Programs and sexual and reproductive health, and coordinate local and international (UNAIDS, WHO, UNFPA, UNICEF, the Global Fund) advocacy. The current state of policy guidelines and our study underscores that this is still an important call to action, and that overcoming historical program silos will require a concerted effort.

Stakeholders discussed the need for more central control of integrated HIV/FP services as a means of ensuring quality and consistency. Perhaps in response to these concerns, the recently published 2015 Rwandan Health Sector Policy guidance (which continues to promote the Government of Rwanda's decentralization policy adopted in 2001) describes the centrally created “Technical Working Groups” established partly to facilitate discussion of programs, performance results, and revising targets between central stakeholders and district‐level implementers.^36^

Primary limitations to this study concern generalizability and sample size. Results from in‐depth interviews are usually not considered generalizable on their own because of small sample sizes and nonrandom sampling methods.^16^ However, the utility of in‐depth interviews is to generate valuable information for programs and/or policies to be used in conjunction with a larger evidence base. Sample size questions for such qualitative studies are often a concern, and no hard cutoffs exist for determination of sample sizes. The general rule is to use “theme saturation” as the guiding principle for sample size, and to stop recruitment after similar topics, themes, and issues emerge from the interviewees, which can occur with as few as 6 interviews.^16^

Evidence‐based models for integrated FP and HIV services have been called for by international stakeholders.^5^, ^6^, ^7^ The ultimate goal of our work was to build consensus and secure political commitment for an integrated model of HIV and FP service provision. An integrated model focused on couples and LARC was supported by Rwandan stakeholders in our study. After this study, coauthors SA and EK met with the acting health office director and the FP and reproductive health specialist at USAID to advocate for LARC promotion and integrated HIV/FP services for couples. Rwanda Zambia HIV Research Group leadership continues to meet with the MoH FP Technical Working Group to discuss results of this work and RZHRG's continued experiences in LARC promotion, CVCT provision, and service integration. The MoH FP partners have subsequently stated support and committed to future collaboration to advance policies on HIV/FP services with a focus on LARC and inclusive of couples. Recommendations to support these policy advancements include inclusion of HIV/FP service integration focusing on LARC methods on agendas of important technical working groups and policymaking forums, continued collection and dissemination of data needed to support policy change, and continued stakeholder pledges of commitment and resources for evidence‐based action.

## CONFLICT OF INTEREST

The coauthors have no conflicts of interest.
